# Evaluating the Effectiveness of Virtual Fracture Clinics in Managing Toe Fractures: Patient Demographics and Outcomes

**DOI:** 10.7759/cureus.70555

**Published:** 2024-09-30

**Authors:** Abdullah Bin Sahl, Faaris Z Niaz, Upamanyu Nath, Umar Afzal, Muhammad Usman, Tom Collins, Anand Pillai

**Affiliations:** 1 Trauma and Orthopaedics, Wythenshawe Hospital, Manchester University NHS Foundation Trust, Manchester, GBR; 2 Trauma and Orthopaedics, Royal College of Surgeons in Ireland, Dublin, IRL; 3 Trauma and Orthopaedics, University of Manchester, Manchester, GBR; 4 Emergency Medicine, Royal Blackburn Teaching Hospital, Blackburn, GBR; 5 Trauma and Orthopaedics, Royal Oldham Hospital, Oldham, GBR

**Keywords:** faam (foot and ankle ability measure), foot and ankle fracture, foot injury, orthopaedic, orthopaedic department, sports medicine, toe fracture, toe injury, vfc, virtual fracture clinic

## Abstract

Background

Virtual fracture clinics (VFCs) offer a remote alternative to traditional fracture clinics, potentially reducing the burden on in-person services. This is particularly relevant for fractures that typically do not require follow-up, such as toe fractures, which are commonly managed conservatively.

Methods

This study evaluated the effectiveness of VFCs in managing toe fractures. Patients treated conservatively were identified using HIVE software, and their outcomes were assessed via a telephone survey. The survey included the Foot and Ankle Ability Measure (FAAM) and additional questions about their recovery process.

Results

Among 56 respondents (62.5% male, median age 26.0 years), the majority sustained injuries to the right foot (62.5%), great toe (71.4%), and proximal phalanx (67.9%). Patient satisfaction was high, with 92.9% reporting a positive experience and 98.2% finding the advice provided helpful. Median FAAM scores were 100% across both subscales, with a median recovery time of 6.0 weeks. Despite high satisfaction and favorable clinical outcomes, factors such as female gender and increased age were linked to poorer outcomes.

Conclusion

VFCs demonstrate high patient satisfaction, positive clinical outcomes, and cost-efficiency, making them a viable alternative to traditional fracture clinics, particularly for conservatively managed fractures. Further research should involve larger sample sizes, prospective study designs, and control groups to validate these findings.

## Introduction

Traditionally, patients presenting with non-emergent bone injuries are scheduled for follow-up appointments in fracture clinics after discharge from the Accident and Emergency (A&E) department. However, this model has been critiqued, particularly for cases such as toe fractures, which often do not require further evaluation post-discharge. This can lead to nonattendance, as some patients may perceive these appointments as unnecessary [1]. A study conducted in central London [2] revealed that phalangeal fractures, comprising 9% of new referrals to fracture clinics, were associated with a 19% nonattendance rate, contributing to inefficiencies and increased costs within the healthcare system.

These issues may be alleviated through the implementation of Virtual Fracture Clinics (VFCs). Unlike traditional face-to-face appointments, VFCs facilitate the remote review of patients, offering advice and determining the necessity of in-person follow-up. For instance, a fracture clinic at King’s College Hospital NHS Foundation Trust reported that following VFC reviews, 48% of patients required no further face-to-face consultations [3]. The introduction of the VFC model was also associated with reduced waiting times for face-to-face clinics due to virtual triaging and ensured that referrals or in-person appointments were arranged in a timely manner relative to the injury [3].

Toe fractures are notably prevalent, with an annual incidence of up to 40 per 100,000, making them the second most common foot injury and accounting for 3.6%-8% of all injuries [4-8]. Despite their frequency, most phalangeal fractures are managed conservatively without the need for surgical intervention [9]. Treatment typically involves immobilizing the digit, often through buddy-taping the affected toe to adjacent ones and using a rigid-sole shoe, although casting may be necessary in some cases. This conservative management, which also includes icing, elevation, and analgesia, generally results in excellent outcomes and high patient satisfaction [10]. The most common mechanisms of injury include axial forces, such as stubbing the toe, or crushing injuries from heavy objects [9].

This study aims to assess the effectiveness of VFCs, focusing on the outcomes and satisfaction of patients undergoing conservative treatment for toe fractures. Additionally, the study will examine the demographic characteristics of the patients included in this VFCs cohort of toe fractures.

## Materials and methods

Patient information

Patients who received conservative treatment for phalangeal fractures between January 1st, 2022, and April 21st, 2024, were identified through VFC patient records using the HIVE software, the electronic patient record system of our acute hospital. Demographic information, including age, gender, and pertinent comorbidities such as diabetes and smoking status, was collected. Additional data gathered included the fracture location, mechanism of injury, and details of the patient’s current medical management plan.

The process of initial fracture management involved patients receiving treatment in the ED, where they underwent X-rays to confirm the fracture diagnosis. This typically included analgesia and splinting. Those associated with open wounds were washed, and nail bed and wound repair were performed in the ED if necessary. Patients presenting with isolated phalangeal fractures were given a wedge boot to remove pressure from the forefoot (Darco Orthowedge Healing Shoe Square Toe boot) and advised on follow-up care (VFC if other injuries were excluded). Isolated great toe fractures were managed with a toe spica splint. More complex injuries, such as multiple phalangeal fractures with metatarsal injuries or crushed foot injuries, were first discussed with the on-call orthopedic team to rule out associated Lisfranc injuries. If a Lisfranc injury was excluded after review, the patient was referred to the VFC. Confirmed or suspected Lisfranc injuries were either referred for a face-to-face orthopedic outpatient review or considered for hospital admission.

Patients referred to the VFC with phalangeal fractures were contacted by phone and invited to participate in a survey. Respondents were asked to provide information regarding their recovery times, defined as the period until they became asymptomatic, including any residual swelling or pain. They were also asked to complete a series of questions designed to generate a Foot and Ankle Ability Measure (FAAM) score.

Measures of outcome

The FAAM, a self-reported instrument published in 2005 in Foot and Ankle International, was used to assess physical function in individuals with musculoskeletal disorders of the leg, ankle, and foot [11]. The FAAM comprises two subscales: the Activities of Daily Living (ADL) Subscale and the Sports Subscale. The ADL Subscale includes 21 scored items related to basic functions of the foot, such as standing, walking on various surfaces, walking barefoot, stair navigation, personal care, and recreational activities. The Sports Subscale, which consists of 7 scored items, pertains to higher-level activities like running, jumping, and landing, and is only applicable to athletic individuals.

 Each question assesses the limitation experienced in performing a specific activity, with responses ranging from “No difficulty” to “Unable to do,” yielding scores from 4 to 0 for each item, respectively. Items limited by factors other than foot and ankle issues were marked as “Not applicable” and excluded from scoring. The total scores for the ADL Subscale range from 0 to 84, and from 0 to 28 for the Sports Subscale, with higher scores indicating better function. Given its applicability to a broader range of activities and its frequent use across all patients, the ADL Subscale of the FAAM was primarily utilized for comparative analysis in this study.

The FAAM has been validated as a reliable, responsive, and accurate measure of physical function for foot disorders [11].

Additionally, patients were queried about their experiences with the VFC, including any issues encountered and the perceived helpfulness of the advice provided. They were also asked whether their recovery time exceeded their expectations.

Statistical analysis

Statistical analyses were conducted using the SPSS, version 29. The analyses employed included box plots, scatter plots, and Spearman’s rank correlation tests. A p-value of less than 0.05 was considered statistically significant for the Spearman’s rank correlation test.

## Results

Patient demographics

The survey included 56 patients who received conservative treatment for phalangeal fractures. Of these, 35 (62.5%) were male and 21 (37.5%) were female. The median age of respondents was 26.0 years (IQR: 13.0-67.5), with a mean age of 37.1 years (SD: 27.4). Males had a median age of 16.0 years (IQR: 13.0-53.0) and a mean age of 30.0 years (SD: 23.1), while females had a median age of 64.0 years (IQR: 12.5-74.5) and a mean age of 50.5 years (SD: 30.0). Notably, 26 (46.4%) of the patients were 16 years old or younger.

**Figure 1:**
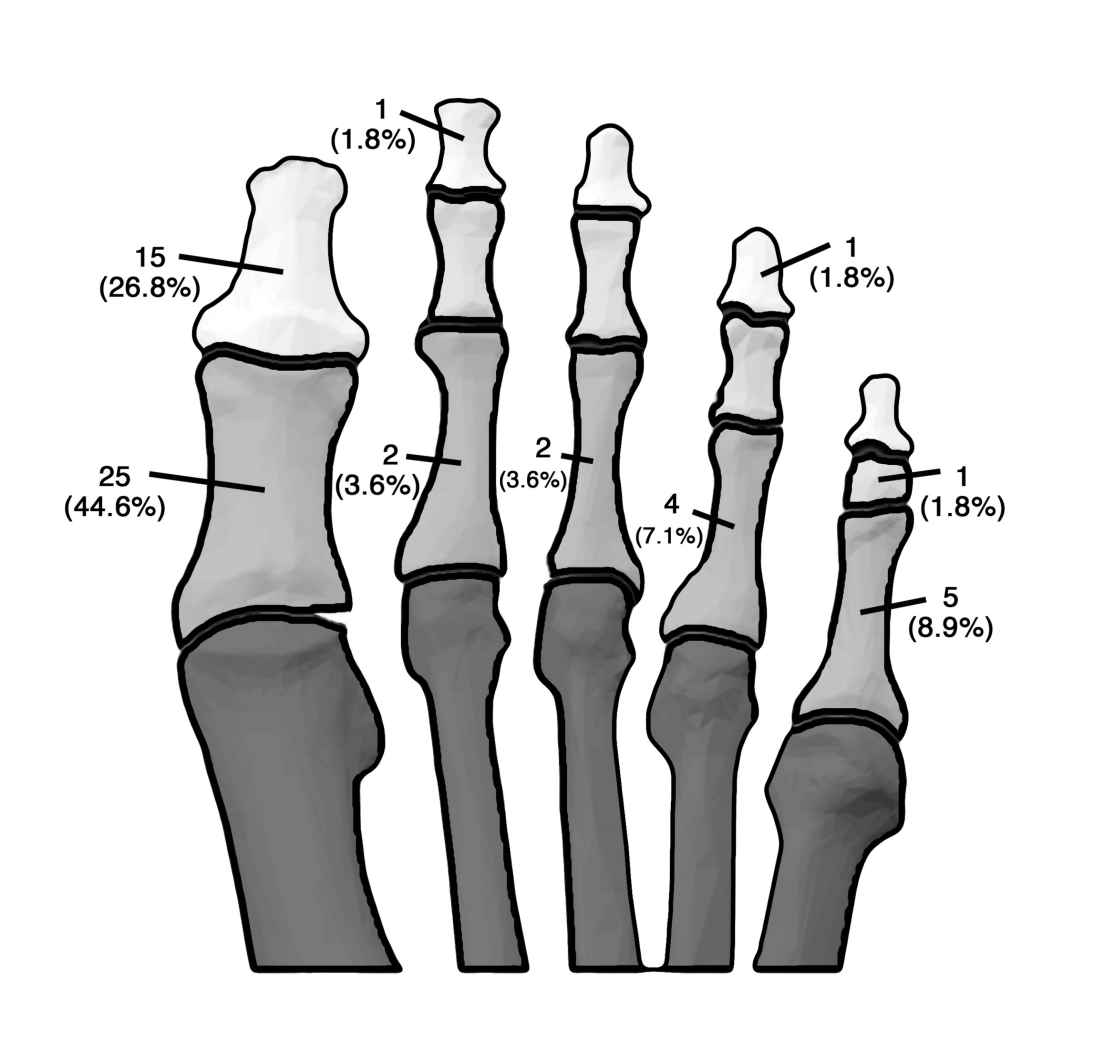
Individual incidence of bone type injured. Source: Sahl AB et al.

Fractures were more common in the right foot (35, 62.5%), with the hallux being the most frequently fractured site (40, 71.4%). The 5th phalanx was the next most common fracture site (6, 10.7%). Fractures of the 2nd, 3rd, and 4th phalanges were less common, occurring in 3 (5.4%), 2 (3.6%), and 5 (8.9%) of cases, respectively. Proximal phalangeal fractures were predominant with 38 fractures (67.9%), followed by distal phalangeal fractures with 17 injuries (30.4%). The right proximal phalanx of the hallux was the most frequently fractured (15, 26.8%). These findings are further labeled discretely in Figure 1.

Among the patients, 2 (6.3%) had diabetes and 7 (12.5%) were smokers. The primary mechanisms of injury were stubbing (47, 83.9%), often related to football or falls, followed by crush injuries (8, 14.3%). One patient did not report any traumatic cause. Closed fractures were most common (53, 94.6%), with only 3 open fractures and 1 displaced fracture observed.

The main treatment was toe spica immobilization (37, 66.1%), followed by buddy taping (16, 28.6%). Other treatments included a boot (2 patients) and a pressure bandage (1 patient), typically administered for 3-4 weeks. Most patients (55, 98.2%) were satisfied with the provided advice, which included rest, ice, elevation, and analgesia.

Patient feedback

A high percentage of patients (52, 92.9%) reported positive experiences with the VFC. Fifty percent of the complaints centered on insufficient communication, such as a lack of follow-up appointments. Other issues included unrealistic recovery expectations (25%) and unclear advice (25%). Additionally, 4 patients (7.1%) experienced slower recovery than anticipated.

Patient outcomes

The ADL Subscale was completed by all 56 patients, while the Sports Subscale was relevant for 33 patients. The median ADL Subscale score was 100.0% (IQR: 99.0-100.0), and the median Sports Subscale score was also 100.0% (IQR: 100.0-100.0). The outcome scores within each respective subgroup are detailed in Table 1.

**Table 1:** ADL outcome scores within each subgroup. ADL: Activities of Daily Living.

			ADL Subscale (%)			Total
			25-50%	50-75%	75-100%	
Gender	Male	Count	0	2	33	35
		% within gender	0.0%	5.7%	94.3%	100.0%
	Female	Count	1	4	16	21
		% within gender	4.8%	19.0%	76.2%	100.0%
Diabetic?	No	Count	1	4	49	54
		% within diabetic?	1.9%	7.4%	90.7%	100.0%
	Yes	Count	0	2	0	2
		% within diabetic?	0.0%	100.0%	0.0%	100.0%
Type	Closed	Count	1	5	47	53
		% within Type	1.9%	9.4%	88.7%	100.0%
	Open	Count	0	1	2	3
		% within type	0.0%	33.3%	66.7%	100.0%
Smoker?	No	Count	1	5	43	49
		% within smoker?	2.0%	10.2%	87.8%	100.0%
	Yes	Count	0	1	6	7
		% within smoker?	0.0%	14.3%	85.7%	100.0%
Mechanism	Stub	Count	1	4	42	47
		% within mechanism	2.1%	8.5%	89.4%	100.0%
	Crush	Count	0	2	6	8
		% within mechanism	0.0%	25.0%	75.0%	100.0%
	No Trauma	Count	0	0	1	1
		% within mechanism	0.0%	0.0%	100.0%	100.0%
Total irrespective of subgroup	Count		1	6	49	56
	% within total		1.8%	10.7%	87.5%	100.0%

Among the 47 patients reporting full recovery, the median recovery time was 6.0 weeks (IQR: 4.0-12.0). Males had a median recovery time of 5.8 weeks (IQR: 4.0-12.0), and females had a median recovery time of 6.0 weeks (IQR: 3.5-12.0). The mean ADL Subscale score was 93.9%, and the mean Sports Subscale score was 96.6%.

The Spearman’s rank correlation coefficient between age and the ADL subscale, inclusive of all patients, was -0.230 with a p-value of 0.089, and the coefficient between age and ADL, exclusive of all patients with an ADL Subscale score of 100%, was -0.631 with a p-value of 0.012. This is shown in Table 2.

**Table 2:** Correlation between age and ADL subscale, shown using Spearman’s rank correlation coefficients. ADL: Activities of Daily Living.

	Spearman’s rho between age and ADL %Subscale	Significance (2-tailed)
Age (inclusive of all individuals)	-0.230	0.089
Age (exclusive of all individuals with ADL subscale score 100%)	-0.631	0.012

## Discussion

Patient demographics

**Figure 2:**
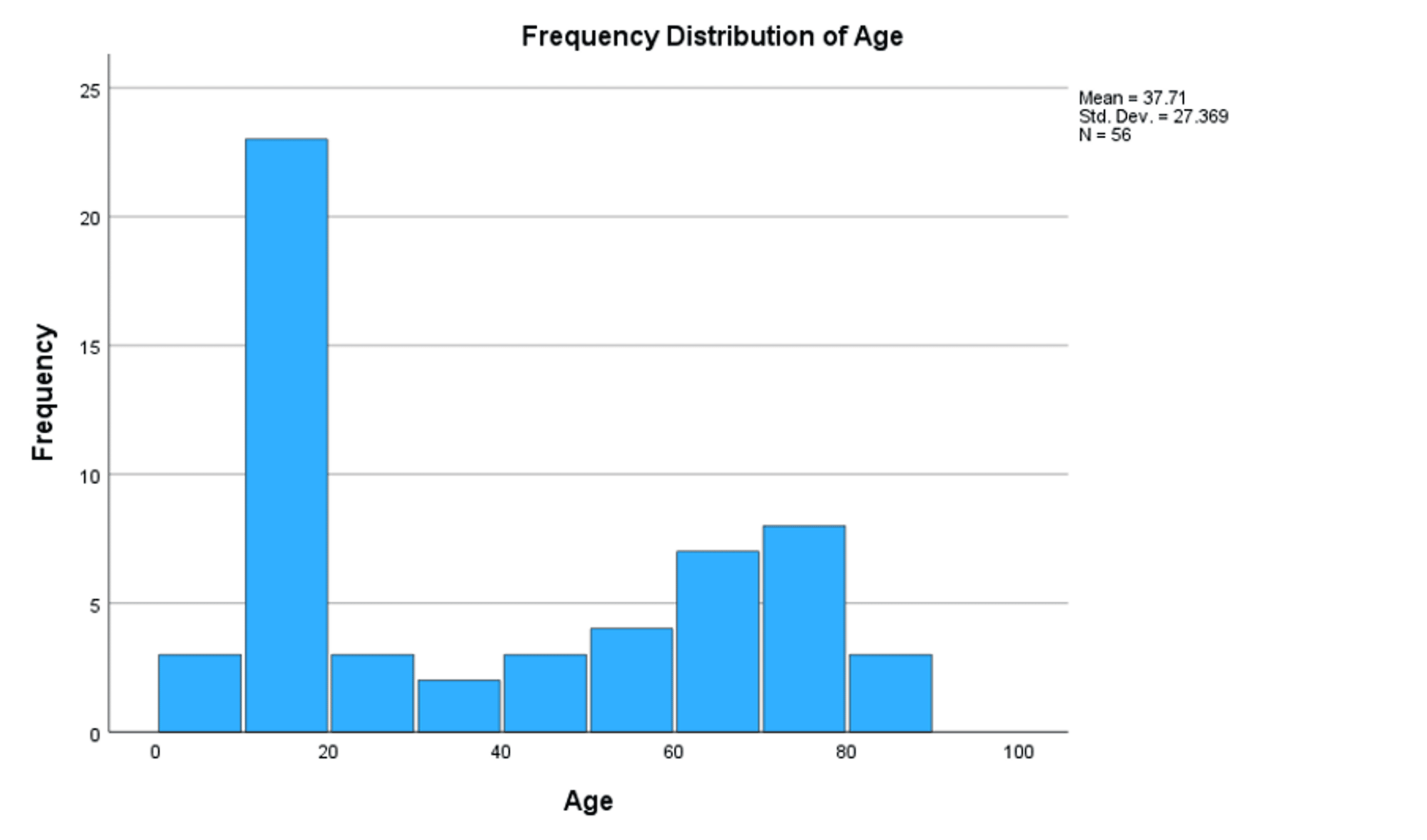
Frequency distribution of age.

The study revealed a male predominance (67%), consistent with existing literature indicating a male-to-female ratio of approximately 1.6 [12]. The median age of 26.0 years was lower compared to other studies, such as Van Vliet-Koppert ST et al.'s median of 35.2 years [10]. This discrepancy may be due to the higher proportion of pediatric patients (46.4% under 16 years), potentially skewing results and introducing response bias from the guardians of younger demographics. However, the mean age in this study was 37.1 years, which is comparable to Court-Brown and Caesar’s mean of 35.3 years [5]. The median age was higher for females (43.3 years) compared to males (26.0 years), aligning with patterns observed in Van Vliet-Koppert ST et al. [10]. The bimodal age distribution, with peaks between 10-20 years and 70-80 years as shown in Figure 2, likely explains the disparity between the median and mean ages, as the median was influenced by the large number of younger patients, whereas the mean reflects the older age groups.

The prevalence of comorbidities in our study was lower than reported in the literature [10], with 2 (3.6%) patients having diabetes and 7 (12.5%) smoking, possibly due to the high proportion of pediatric patients. Fracture site distribution was consistent with other studies, such as that by Eves and Oddy [2], showing the hallux as the most commonly fractured site, followed by the 5th, 4th, 2nd, and 3rd phalanges. The high percentage of hallux fractures (71.4%) compared to the 17-24% range reported elsewhere [13] may be due to the overrepresentation of pediatric patients, who are more prone to such injuries [10]. However, even within this subgroup, hallux fractures are still overrepresented, with other studies like Van Vliet-Koppert ST et al. finding that great toe fractures made up 41.9% of patients younger than 16 years [10], compared to 79.2% in our study.

Patient feedback

Patient satisfaction with the VFC was high, with 92.9% of patients reporting no complaints and 98.2% finding the advice helpful. This is consistent with other studies showing high satisfaction with VFCs, where 77.0% of patients were content with remote management [14] and 81.0% reported satisfaction [15]. For conservative management of toe fractures, Van Vliet-Koppert ST et al. also reported high satisfaction with a median visual analog scale score of 10/10, although the study did not specify whether a VFC model was used [10].

Patient outcomes

The study found excellent clinical outcomes, with median FAAM scores of 100% for both the ADL and Sports Subscales. Mean scores were 93.9% for the ADL Subscale and 96.6% for the Sports Subscale, indicating overall good recovery. The higher mean score for the Sports Subscale may reflect the inclusion of more active patients. No patients reported a recovery below 25%, and only 7.1% experienced slower recovery than anticipated. Van Vliet-Koppert ST et al. reported similar outcomes with a different metric, the American Orthopaedic Foot and Ankle Society (AOFAS) midfoot score, showing a median score of 45/45 [10]. The low percentage of patients reporting residual pain (1.3%) in a systematic review by Davey MS et al. [15] aligns with the favorable FAAM outcomes observed in this study.

The median recovery time for full recovery was 6.0 weeks, with a mean of 8.1 weeks, aligning with NHS guidelines that suggest a typical recovery period of 4 to 6 weeks [16] and comparable to recovery times reported by Robertson GA et al. [17]. It is important to note that the study's recovery time metrics may not include patients who had not yet fully recovered at the time of the study, potentially underestimating recovery times.

Outcomes by demographic factors

**Figure 3:**
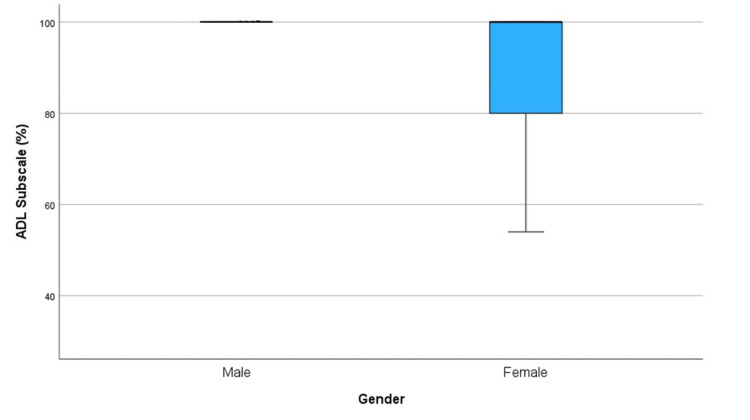
Box plot to compare ADL Subscale scores (%) between males and females. ADL: Activities of Daily Living.

Analysis by demographic factors revealed a weak negative correlation between age and ADL Subscale scores (Spearman’s Rank Correlation coefficient of -0.23, not significant at the 0.05 level). However, for patients who did not achieve full recovery, the correlation was stronger and statistically significant at -0.631 (p = 0.012). The negative slope correlation can also be observed in scatter plot Figure 3. This suggests that while most patients across all age ranges achieved full recovery, older patients who did not recover fully had poorer outcomes. In other words, in the group of patients that do not recover fully, older patients tend to have poorer outcomes. The study by Van Vliet-Koppert ST et al. comparatively did not find age to be a significant factor in outcome scores, possibly due to overall high outcome scores in their study [10]. However, other studies such as Egol KA et al., who studied ankle fractures, have found that older age was a predictor of poorer recovery [18].

**Figure 4:**
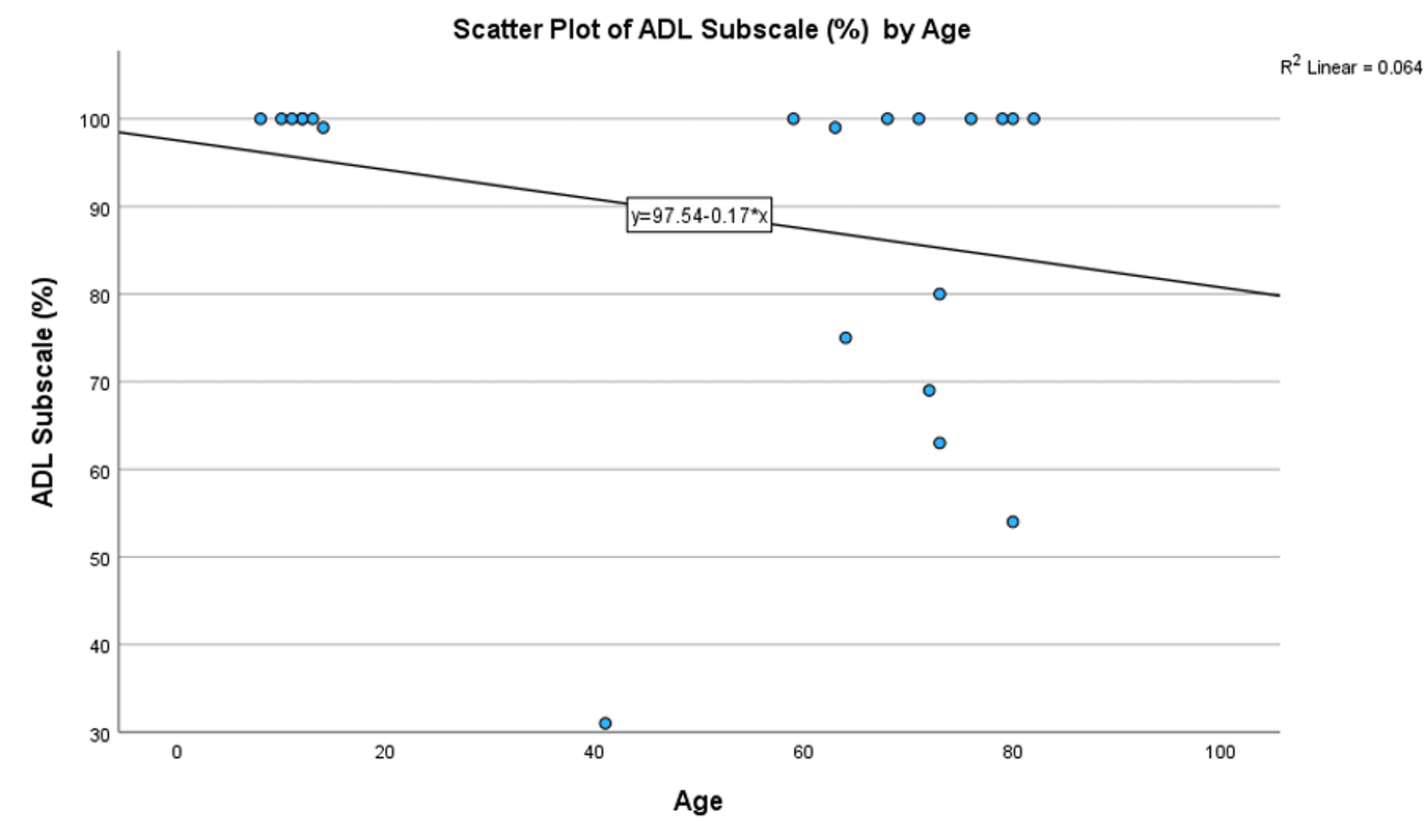
Scatter plot of ADL Subscale scores (%) by age of female patients. ADL: Activities of Daily Living.

**Figure 5:**
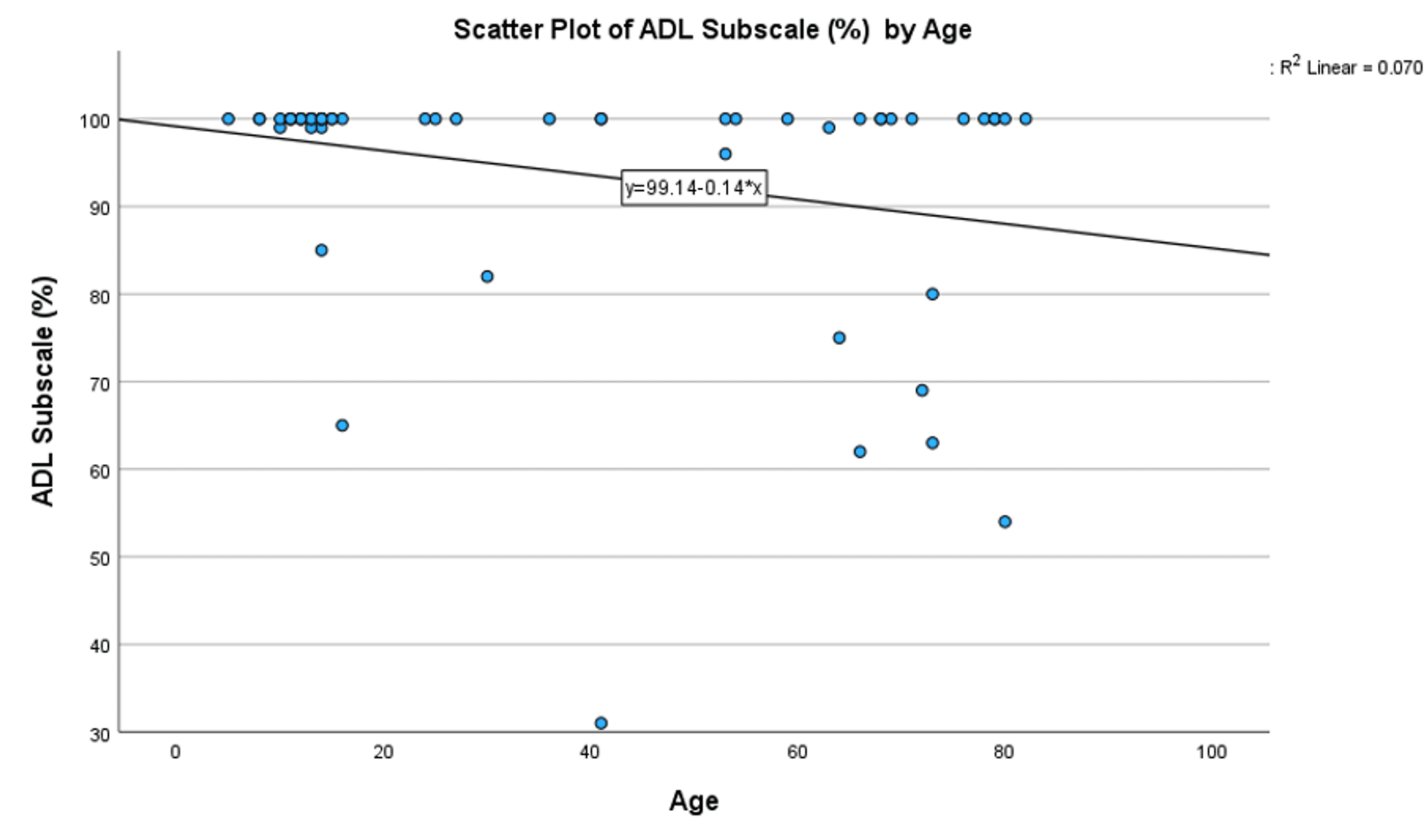
Scatter plot of ADL Subscale scores (%) by age. ADL: Activities of Daily Living.

For gender, the trend between age and ADL outcomes was more pronounced in female patients, who had lower minimum and first quartile ADL scores compared to males. The analysis revealed that a significantly higher proportion of female patients (23.8%) scored between 25-75% on the ADL Subscale compared to male patients (5.7%), as shown in Table 1. Notably, the only patient in the study who scored below 50% was female. This is further illustrated in the box plot, Figure 3, which shows that female patients had both a lower minimum (non-outlier) value and a lower first quartile value than their male counterparts. While the study by Van Van Vliet-Koppert ST et al. [10] found no direct association between gender and outcome as measured by the AOFAS score, it did indicate that younger female patients reported lower satisfaction with their outcomes. This disparity in satisfaction may stem from the AOFAS score's lower sensitivity compared to the VAS in detecting certain impairments as noted in their study. These findings suggest that female gender may be associated with poorer outcomes, a conclusion supported by prior studies that have demonstrated worse outcomes for female patients with ankle fractures [18]. This stronger correlation can be further demonstrated in Figure 4 compared to Figure 5, indicating poorer outcomes specifically for older females. The observed poorer outcomes in females may also reflect age-related confounding, given the higher median age of female patients in our study.

**Figure 6:**
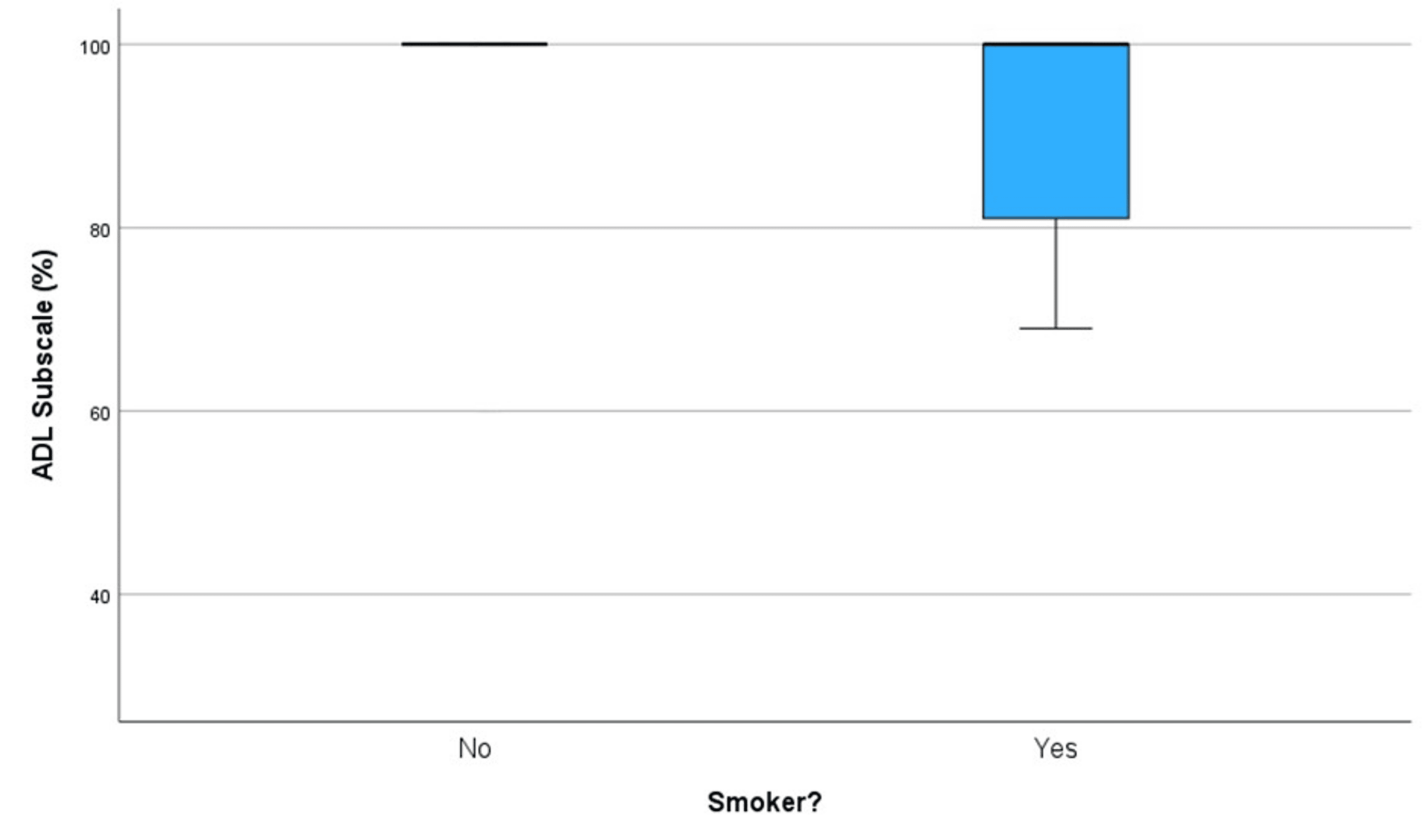
Box plot to compare ADL Subscale scores (%) between smokers and non-smokers. ADL: Activities of Daily Living.

Diabetes or smoking was associated with poorer outcomes in our study. However, this should be interpreted with caution due to the small number of diabetic patients (two patients) and smokers (seven patients) limiting the robustness of their association. It may be worth noting that of the two diabetic patients, neither made a full recovery (75% and 63%) according to their ADL Subscale score as shown in Table 1. A box plot graph in Figure 6 compares the outcomes between smokers and non-smokers, showing that smokers had a lower minimum (non-outlier) value and a lower first quartile ADL Subscale %.

**Figure 7:**
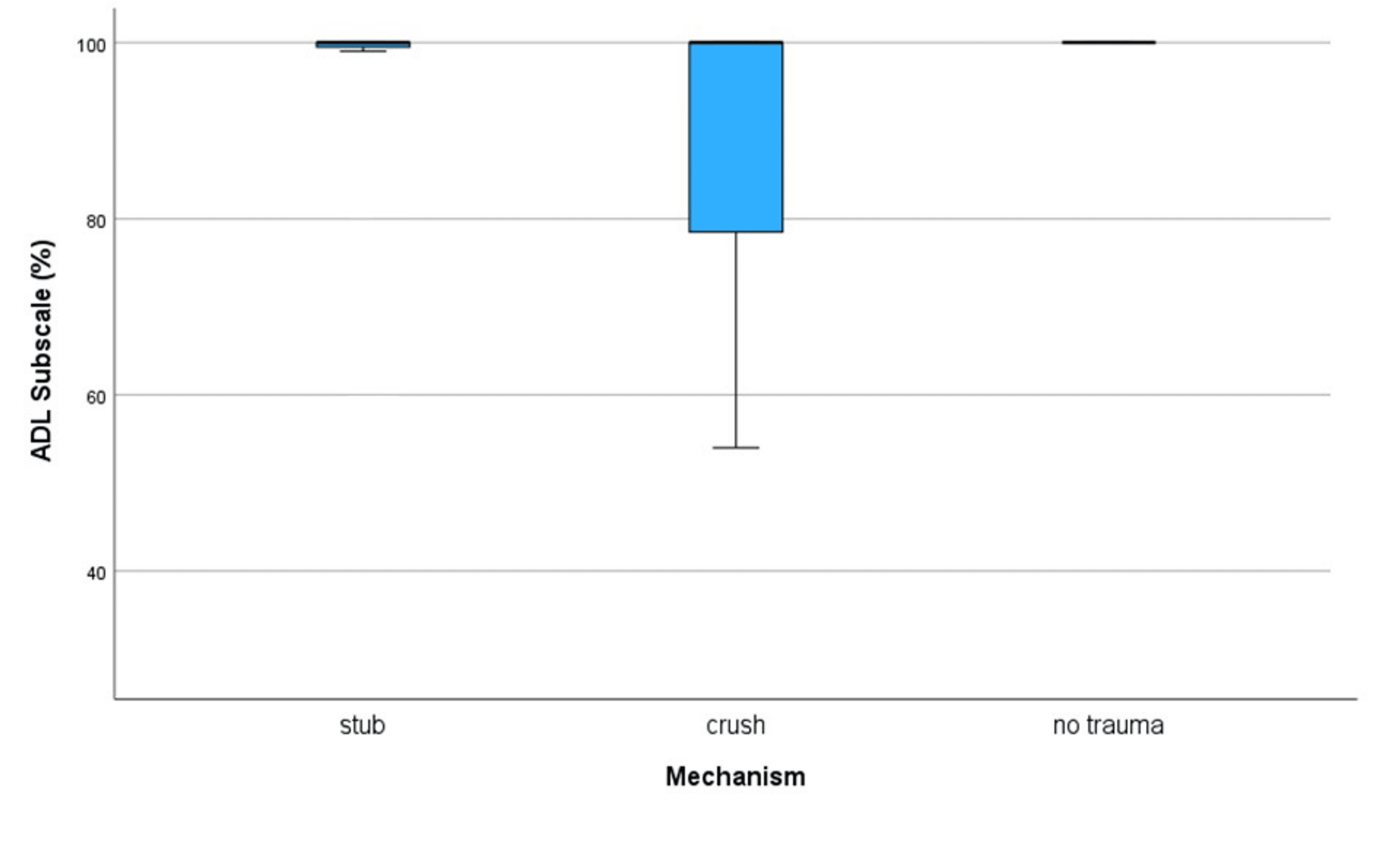
Box plot to compare ADL Subscale scores (%) between mechanisms of injury to toes. Note that the “no trauma” group consisted of just one patient, and analysis of this group is therefore limited. ADL: Activities of Daily Living.

Fractures resulting from crush injuries were associated with poorer outcomes compared to stub injuries, potentially due to the higher incidence of open fractures among crush injuries (Figure 7). Open fractures in this study were linked with lower recovery outcomes, consistent with previous findings that open fractures generally have poorer outcomes compared to closed fractures [19]. Among the three patients with open fractures, only one achieved a full recovery, as indicated by a 100% score on the ADL Subscale. Furthermore, 33.3% of these patients had a recovery outcome below 75%, as demonstrated in Table 1. The mean ADL Subscale score for this group was 90.3%, notably lower than the overall average observed across the study.

Efficiency of VFCs

The VFC model meets the British Orthopaedic Association Standards for Trauma (BOAST) guidelines, which advise that patients should be seen within 72 hours [20, 21], with patients seen within an average of 1.6 days [14]. This study confirms that the VFC meets other BOAST guidelines, including the ability to refer patients directly to physiotherapy [20]. VFCs have been shown to be more cost-effective, with an average cost of £71 per patient compared to £124 for traditional clinics [15]. VFCs reduce the need for in-person visits, freeing up resources for face-to-face appointments and addressing high non-attendance rates for toe fracture follow-ups, which can be as high as 19% [2]. Advice given should cover pain duration, the timeline for avoiding contact sports, and the use of protective footwear, all of which can be effectively communicated virtually without necessitating a clinic visit [2]. In our study, all patients were given advice on rest, elevation, ice, and analgesia in the VFC appointment, with all patients also feeling that they had the opportunity to ask additional questions if needed during the VFC appointment. The VFC model, therefore, allows for effective virtual management and advice, consistent with the increasing adoption of VFCs across the UK and Ireland [15].

Limitations

This study has several limitations, including a small sample size (56 patients) and a skew towards younger patients, which may not represent the general demographic of toe fractures. The retrospective design and reliance on self-reported data and hospital notes introduce potential biases. The use of telephone surveys may have introduced response and social desirability biases. The study's focus on conservative management of toe fractures limits generalizability, and the absence of a control group restricts comparative analysis. The ceiling effect of the FAAM score of 100% may have limited sensitivity in detecting minor differences among higher-scoring patients (this can be visualized in the box plots Figures 3, 6, and 7).

Recommendations for future studies

Future research should involve larger sample sizes to enhance representativeness and reduce demographic skew. A prospective study design will also improve data accuracy and reduce recall bias. Including a control group for comparison between the VFC and traditional fracture clinics will provide more robust insights. Additionally, an evaluation of the costs of VFCs can be explored in greater detail to demonstrate whether a VFC model will actually reduce costs. It will be advantageous to broaden the focus beyond conservatively managed toe fractures and incorporate a more diverse patient cohort, to enhance the robustness of the research. Additionally, employing a wider array of outcome measures beyond FAAM, such as the AOFAS mentioned previously, can be beneficial. This latter approach may help mitigate the ceiling effect seen with FAAM by introducing additional criteria that better distinguish participants who achieve higher scores.

## Conclusions

This study highlights the effectiveness of VFCs in managing toe fractures conservatively. The study demonstrated favorable clinical outcomes, as evidenced by FAAM scores and recovery times, alongside high patient satisfaction rates with the VFC. These results are consistent with prior research on the effectiveness of VFCs. The demographic analysis in our study shows a younger, predominantly male cohort with a high incidence of hallux fractures. It is noted that this outcome may have been influenced by the limited sample size and potential biases inherent in the study. Female patients experienced less favorable outcomes, and recovery was significantly poorer with age among the individuals who did not recover fully. Diabetes, smoking, and open fractures were linked to poorer results; however, these associations were limited by the small sample sizes.

Overall, VFCs are a promising alternative to traditional fracture clinics, offering efficient management and satisfactory results. Future research can include larger samples, control groups, and multiple outcome measures to validate these findings and explore the broader applicability of VFCs.

## Figures and Tables

**Figure 8:**
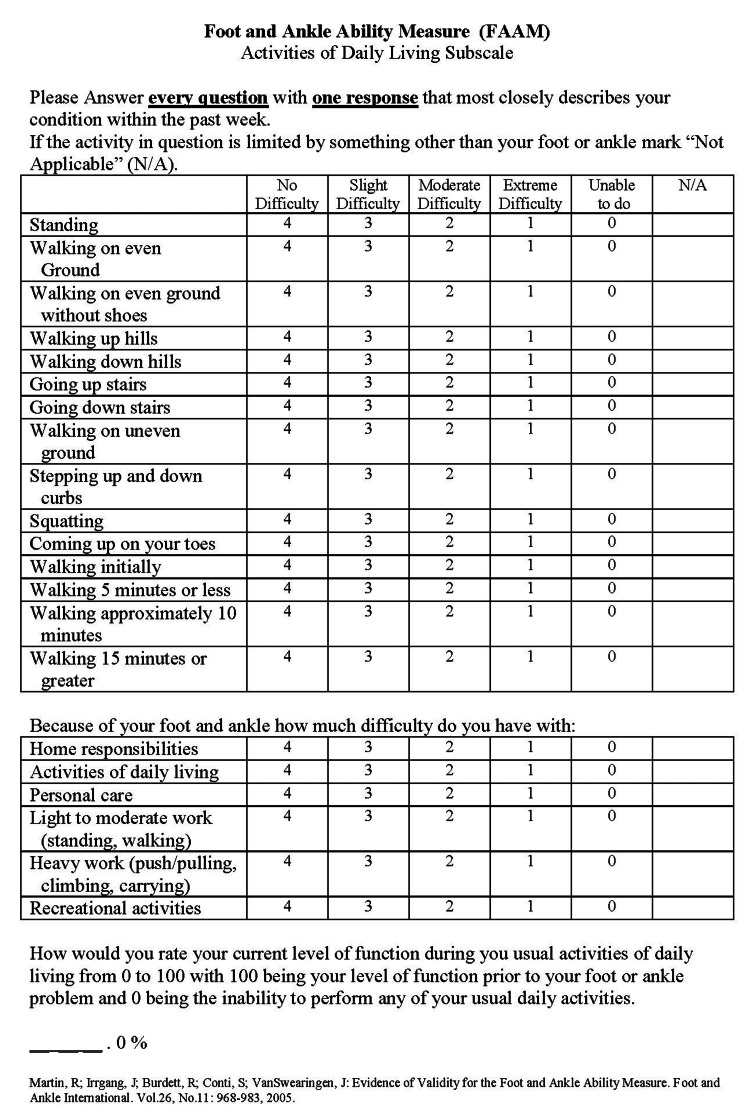
Foot and Ankle Ability Measure Page 1. Reference: [11]

**Figure 9:**
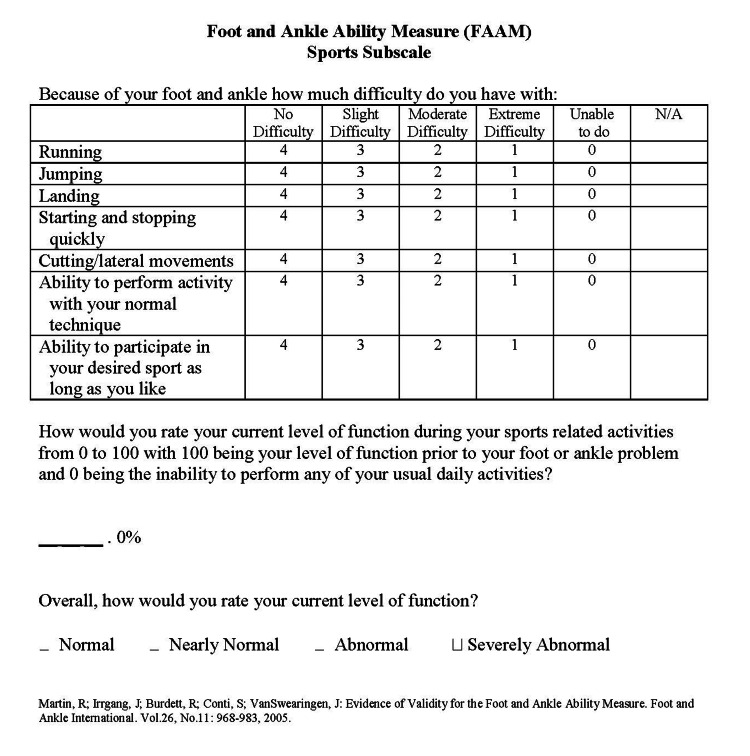
Foot and Ankle Ability Measure Page 2. Reference: [11]
